# Comparison of Class II Bulk-Fill, Self-Adhesive Composites, Alkasite, and High-Viscosity Glass Ionomer Restorations in Terms of Marginal and Internal Adaptation

**DOI:** 10.3390/ma17174373

**Published:** 2024-09-04

**Authors:** Agnès Sahli, Laurent Daeniker, Isaline Rossier, Luciana Caseiro, Enrico di Bella, Ivo Krejci, Tissiana Bortolotto

**Affiliations:** 1 Division of Fixed Prosthodontics and Biomaterials, University Clinic of Dental Medicine (CUMD), University of Geneva, 1205 Geneva, Switzerland; 2 Division of Cariology and Endodontology, University Clinic of Dental Medicine (CUMD), University of Geneva, 1205 Geneva, Switzerland; laurent.daeniker@unige.ch (L.D.); isaline.rossier@unige.ch (I.R.); luciana.caseiro@unige.ch (L.C.); enrico.dibella@unige.it (E.d.B.); ivo.krejci@unige.ch (I.K.)

**Keywords:** alkasite, glass ionomer, bulk-fill, self-adhesive composite, marginal adaptation, internal adaptation, pedodontics

## Abstract

(1) Background: Restoring decayed teeth in young patients can be challenging. This calls for a simplification of the protocols through new biomaterials. Therefore, the aim of this study was to compare the marginal adaptation delivered by restorative materials applied on class II cavities by using a simplified protocol, before and after fatigue test, followed by the assessment of the internal adaptation. (2) Methods: Forty-eight human teeth were divided into six groups (n = 8). Dentinal fluid simulation was performed before restoring the class II cavities: Gr 1—adhesive (Clearfil Universal Bond Quick) and nanohybrid flowable composite (Clearfil Majesty ES Super Low Flow), Gr 2—adhesive (Clearfil Universal Bond Quick) and nanohybrid composite (Clearfil Majesty ES standard), Gr 3—bulk fill self-adhesive composite (Surefil One), Gr 4—bioactive powder-liquid filling material (Cention Forte), Gr 5—universal adhesive (Adhese Universal) and nanohybrid composite resin (Tetric Powerfill); and control group (CT)—high-viscosity glass ionomer (Equia Forte). Marginal adaptation was observed with scanning electron microscopy (SEM) and compared before and after a fatigue test consisting of repeated thermal and mechanical cycles. The specimens were then cut mesio-distally, and internal adaptation was undertaken using SEM again. Repeated measures and one way ANOVA followed by a Fisher’s LSD test and Fisher’s LSD post hoc test were used in order to compare the statistically significant differences among groups. (3) Results: As for the marginal adaptation after loading, Cention Forte (58%) and Equia Forte HT (53%) were statistically equivalent and presented the highest results, followed by Clearfil Majesty ES Standard (32%) and Tetric Powerfill (27%), with Surefil One (8%) and Clearfil Majesty ES Flow Super Low (7%) showing the worst results. In terms of internal adaptation, Cention Forte (85%) and Clearfil Majesty ES Standard (74%) had the highest percentages of continuous margins. Tetric powerfill (56%) and Equia Forte HT (44%) showed statistically significantly lower results, followed by Clearfil Majesty ES Flow Super Low (33%) and eventually Surefil One (17%). (4) Conclusions: This in vitro study showed promising results for the marginal and internal adaptation of alkasite dual cured Cention Forte in the restoration of class II cavities. This material could be considered an interesting restorative alternative for the restoration of deciduous teeth.

## 1. Introduction

Restorative care to achieve oral health in pediatric dentistry requires a global treatment plan including several aspects such as the evaluation of caries risk, the developmental status of permanent teeth, the lifespan of primary teeth, patients’ cooperation, and the durability of dental materials. Regarding the choice of dental materials, amalgam, resin composite, glass ionomer cements, compomers, and stainless-steel crowns have been widely used by dentists advocated to pediatric treatments [1]. 

Resin composite has been successfully rated for the restoration of posterior cavities in primary teeth with annual failure rates in stress-bearing areas of 0–15% in respect to the 0–26% of glass ionomers [2]. Despite this superior performance, the drawback of resin composites is their technique sensitivity and a longer restorative procedure, requiring proper isolation from humidity and patient cooperation. In cases in which these technical requirements cannot be fulfilled, resin composite may not be the restorative material to choose for dental treatment in children. 

Glass ionomer, on the other hand, benefits from chemical bonding to Ca from enamel and dentin, a fluoride content that can be released to the restoration surroundings and more tolerance to humidity in respect to resin-based materials. Nevertheless, the major limitation of conventional glass ionomers lies on their poor mechanical resistance, which has been demonstrated in a previous randomized clinical trial with an overall median time from treatment to failure of only 1.3 years when used on stress-bearing areas [2]. 

The need for simplification and to turn restorative procedures more patient friendly has motivated researchers and manufacturers to introduce new biomaterials for the restoration of decayed teeth. Especially for primary teeth, it would be desirable to restore masticatory function and oral health in young patients with a dentist and patient-friendly application protocol by using simplified restorative techniques and easy-to-apply adhesive materials that will last for a limited period of time, i.e., until exfoliation of the decidual tooth occurs.

A range of new materials with specific benefits have been launched into the market. As mentioned above, the purpose while treating young patient is to simplify the protocols. This can be reached through, for example, avoiding the etching step or avoiding the use of an adhesive system, decreasing light curing time, and avoiding the necessity of using a layering technique [3]. 

As a preclinical screening, an in vitro test that simulates intraoral conditions should enable to evaluate the in vitro performance of restorative materials. Moreover, marginal quality is one relevant parameter that is used in calibrated analyses of clinical studies such as USPHS and the FDI clinical criteria [4,5]. As the breakdown of the adhesive interface is considered as the main clinical pattern observed when restorations fail [6], not only marginal but also internal adaptation are important parameters to consider when evaluating the performance of new restorative materials and techniques [7].

For the above-mentioned reasons, the purpose of this study was to compare the marginal adaptation delivered by restorative systems, i.e., adhesive/coat and restorative material from the same manufacturer, applied on class II cavities by using a simplified protocol, before and after a fatigue test consisting of repeated thermal and mechanical cycles, followed by the assessment of the internal adaptation to determine if gaps at restoration margins could propagate inside the cavity and degrade the internal adhesive interface as well. The testing groups would consist of a micro hybrid resin composite in flowable and standard versions, applied in one single layer combined with a universal adhesive in self-etching mode; a bulk filling resin combined with a universal used in self-etching mode; a self-adhesive composite; a dual-cure resin based material containing alkaline combined with a self-curing adhesive contained in the dedicated applicator; and a high-viscosity glass ionomer cement combined with a self-adhesive light curing resin coat.

The null hypotheses tested were that no difference would be detected in both marginal and internal adaptation of restorations of class II cavities with the different materials applied by using a simplified application protocol.

## 2. Materials and Methods

The restorative materials chosen to be analyzed in the test groups were a light-cure, universal composite in flowable and standard versions (Clearfil Majesty ES Low Flow and Standard), a bulk fill self-adhesive composite (Surefil One), a bioactive powder-liquid dual cured filling material (Cention Forte), and a bulk fill light cure composite (Tetric Powerfill). For the control group, a glass ionomer (Equia Forte) was selected. The list of materials, their composition, and their main properties are detailed in Table 1.

Sixty human teeth that were intact, with a similar size and presented no carious lesion, were selected. They were obtained in accordance with the local institution guidelines, anonymously collected and therefore not requiring ethical committee approval, and they were stored in 0.1% thymol solution under refrigeration until being used for the study. After cleaning these teeth with a scaler and toothpaste, they were prepared for dentinal fluid simulation. In order to do so, the apices of the roots were sealed with an adhesive system (Optibond FL, Kerr, Brea, CA, US) and fixed on alumina holders—as those used for scanning electron microscope (SEM) with a resin composite (Tetric, Ivoclar Vivadent, Schaan, Liechtenstein) and surrounded with a cold polymerizing resin (Technovit 4071, Heraeus Kulzer, Hanau, Germany). To simulate intratubular fluid flow, a cylindrical hole was drilled under the cemento-enamel junction of the crowns, on the buccal side, and 1.2 mm diameter metallic tubes were inserted and fixed using an adhesive system (Optibond FL) and composite resin (Tetric). Twelve of the teeth were used in order to define the cavity size and as pretests. For the forty-eight remaining teeth, the connection between the pulp chamber and the simulated intratubular fluid flow (3:1 ratio of phosphate-buffered saline/diluted horse serum (Bioswisstec AG, Schaffhausen, Switzerland) under a hydrostatic pressure of 25 mm Hg was established with a flexible silicone hose inserted in the metallic tubes on the one side and in a bottle containing the fluid, placed 34 cm above the samples in the other side, following the protocol described in detail by Krejci et al. (1993) and Bortolotto et al. (2007) [8,9]. This preparation was maintained during preparation, restoration, and loading of the specimens. Two pre-test teeth were used for determining the final dimensions of cavity preparations. In order to imitate pedodontic restorations in terms of dimensions, the cavity size of class II cavities was 4.5 mm in length and 3 mm in height at the interproximal level, and 1.5 mm deep in occlusal, following the natural grooves of the teeth. Forty-eight teeth were divided into five test groups and one control group. An adhesive system (Clearfil Universal Bond Quick) was applied according to the manufacturer’s instructions, i.e., stored less than seven minutes in the mixing plate, applied with microbrush, no waiting time required, drying for more than five seconds until the bonding agent did not move, and with polymerization for ten seconds with a curing device in the two first test groups. 

For the first one (1), an adhesive (Clearfil Universal Bond Quick) was applied, followed by a light-cure, universal flowable composite (Clearfil Majesty ES Flow Super Low) in one layer, and polymerized for twenty seconds. In the second group (2), the same protocol was followed with the standard version of the same restorative material (Clearfil Majesty ES standard). 

The third test group (3) was restored with a bulk fill self-adhesive composite (Surefil One) following the manufacturer’s recommendations—mixing of the cap for 10 s, 4200–4600 osc/min, 1 min 30 s working time, light polymerization for 20 s, and 6 min waiting time before taking out the matrix to let the chemical curing take place in depth. 

The fourth test group (4) was restored using a bioactive powder-liquid dual cured filling material (Cention Forte) according to the manufacturer’s recommendations—mixing of a drop of Cention Primer with the applicator in the mixing plate for five seconds, applying in the cavity for 10 s, blowing, activating the Cention Forte cap, mixing in the machine for 17 s, allowing a working time of 20 s, and performing polymerization with a curing device (Bluephase PowerCure, Ivoclar Vivadent, Schaan, Liechtenstein) for 2 × 5 s in mode T (power density of 1800–2200 mW/cm^2^). 

A bulk fill light cure composite (Tetric PowerFill) was used to restore the last test group (5), also following the manufacturer’s instructions—Adhese Universal was applied for 20 s, the mixture was air blown and then polymerized with Bluephase Power Cure for 3 s, and following this, Tetric Powerfill was applied in one layer and the mixture was polymerized for 3 s with the same lamp. 

All restorations were carefully polished using red and white ring diamond burs (Intensiv SA, Montagnola, Switzerland), followed by rubber points (brownie and greenie, Shofu, San Marcos, CA, USA) and flexible discs (SofLex Pop-On, 3M ESPE, St. Paul, MN, USA) under 12× magnification. 

A high-viscosity glass ionomer (Equia Forte HT) was used for the control group (C). In order to apply it in the cavity, the cap was shaken, then activated and mixed for 10 s (working time 1 min 30 s), applied in one layer and let to set for 2 min 30 s after mixing. Equia Forte coat was dispensed and polymerized during 20 s with a light-curing device after following the same polishing protocol as the test groups.

After polishing the class II restorations, the sample’s replicas with a polyvinylsiloxane impression material (President The Original—Light body, Coltene, Altstätten, Switzerland) were taken, poured with epoxy resin, and gold coated, and the margins were then analyzed under a scanning electron microscope at 200× magnification by a trained and blinded laboratory technician. Micrographs were taken, and the percentages of continuous margins (%CM) were quantified with a custom-made program. The teeth were then loaded in a chewing simulator CS-8 SD (Meckatronik, Feldkirchen, Germany) for 200,000 load cycles and 500 thermal cycles. After loading, replicas were taken again, marginal analysis of restorations after loading was performed. The specimen was then cut into two mesio-distal slices by using a cutting machine (Accutom-100, Struers, Copenhagen, Danemark) in order to analyze restorations’ internal adaptation. Slices were polished with fine carbide paper, and replicas were taken again with the same polyvinylsiloxane material (President The Original—Light body Coltene). For the evaluation of the internal adaptation, percentages of closed (continuous margins) and open interfaces (non-continuous margins) were quantified as for the marginal adaptation. 

### Statistical Analysis

**Figure 1:**
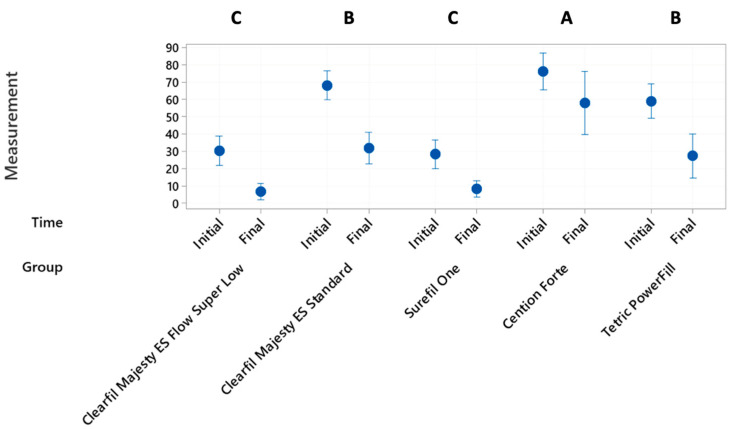
Interval plots of initial and final values per group. Letters A, B and C show the significant differences.

The normal distribution of data enabled the use of parametric tests. The independent variables were the group type before or after loading and the dependent variables were the percentages of continuous margins (marginal adaptation) and percentages of internal adaptation after loading. For the scores of marginal adaptation, differences in marginal adaptation before (initial) and after (final) loading between groups (Figure 1) were assessed by using a repeated measures ANOVA followed by a Fisher’s LSD test.

Grouping information of each group (mean value of initial and terminal %CM, Figure 1) was compared with Fisher’s LSD method and 95% confidence interval.

**Figure 2:**
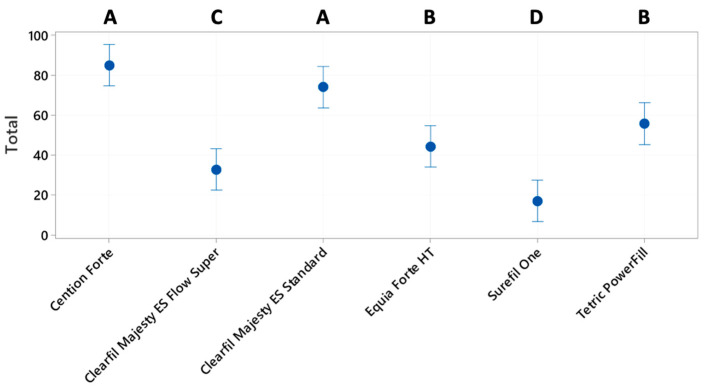
Percentages of continuous margins for marginal adaptation after thermomechanical loading. Letters A, B and C show the significant differences.

The results of marginal %CM after thermomechanical loading for the 6 groups (Figure 2) were compared by a one-way ANOVA followed by a Fisher’s LSD test to check pairwise differences.

**Figure 3:**
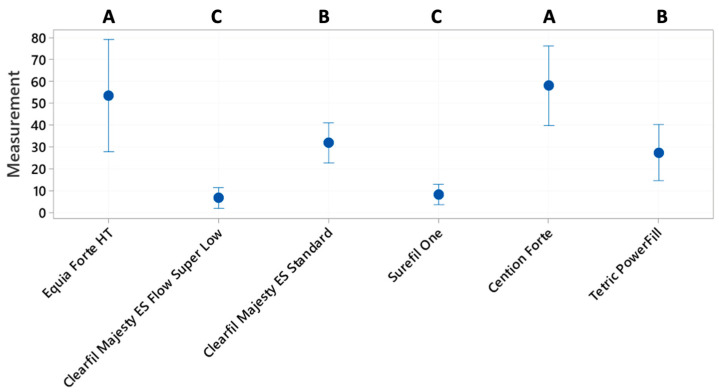
Percentages of continuous margins for internal adaptation after thermomechanical loading. Letters A, B and C show the significant differences.

For the scores of internal adaptation, a one-way ANOVA followed by Fisher’s post hoc test was used to detect statistically significant differences among groups (Figure 3). The level of confidence was set to 95%.

## 3. Results

**Figure 4:**
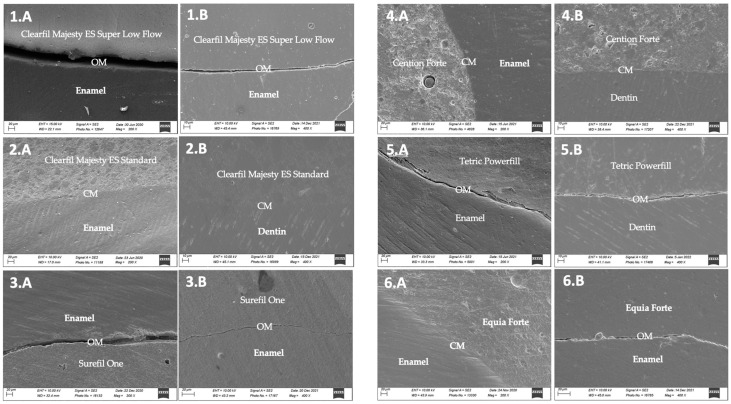
Representative micrographs of different marginal situations observed with the different materials. (**1.A**) Samples from group 1. SEM micrographs of enamel and Clearfil Majesty ES Super Low Flow margins after thermomechanical loading. See the open margins (OM). (**1.B**) Sample from group 1. SEM micrographs of enamel and Clearfil Majesty ES Super Low Flow internal adaptation after thermomechanical loading. See the open margins (OM). (**2.A**) Sample from group 2. SEM micrographs of enamel and Clearfil Majesty ES Standard margins after thermomechanical loading. See the continuous margins (CM). (**2.B**) Sample from group 2. SEM micrographs of dentin and Clearfil Majesty ES Standard internal adaptation after thermomechanical loading. See the continuous margins (CM). (**3.A**) Sample from group 3. SEM micrographs of enamel and Surefil One margins after thermomechanical loading. See the open margins (OM). (**3.B**) Sample from group 3. SEM micrographs of enamel and Surefil One internal adaptation after thermomechanical loading. See the open margins (OM). (**4.A**) Samples from group 4. SEM micrographs of enamel and Cention Forte margins after thermomechanical loading. See the continuous margins (CM). (**4.B**) Samples from group 4. SEM micrographs of enamel and Cention Forte internal adaptation after thermomechanical loading. See the continuous margins (CM). (**5.A**) Samples from group 5. SEM micrographs of enamel and Tetric Powerfill margins after thermomechanical loading. See the open margins (OM). (**5.B**) Samples from group 5. SEM micrographs of dentin and Tetric Powerfill internal adaptation after thermomechanical loading. See the open margins (OM). (**6.A**) Sample from group control. SEM micrographs of enamel and Equia Forte internal margins after thermomechanical loading. See the open margins (OM). (**6.B**) Sample from group control. SEM micrographs of enamel and Equia Forte internal adaptation after thermomechanical loading. See the open margins (OM).

The results of marginal adaptation before/after thermomechanical loading (TML) as well as those of internal adaptation after TML are shown in Figure 1, Figure 2 and Figure 3. One representative SEM image (200×) of each group for both marginal and internal adaptations are presented in Figure 4. 

Statistically significant differences were observed between groups when comparing the percentages of continuous margins (%CM) before/after loading. Figure 1 shows the difference per group before/after thermomechanical loading (initial/final). There was a statistically significant difference between the %CM before/after loading. This meant that all restorations presented marginal degradation after the fatigue test, the least difference before/after when Cention Forte was used as restorative material.

Pooled data of marginal adaptation before/after loading of each group is presented as well in Figure 1. As the %CM before loading for the control group (Equia Forte HT) could not be reported due to the presence of the coating layer that covered restoration margins, the pooled data of initial/final values comparisons of this group was excluded from the analysis (Figure 1, five groups instead of six). Overall, the marginal performance could be grouped into three clusters (A,B,C): Cention Forte (67 ± 20) with the highest results but around 33% of marginal gaps, Clearil Majesty ES Standard (50 ± 21) and Tetric PowerFill (43 ± 21) being equivalent with almost 50% of marginal gaps, and finally Surefil one (18 ± 13) and Clearfil Majesty ES Flow Super Low (18 ± 15) being equivalent with almost 80% of marginal gaps. 

To compare the results of marginal adaptation including the control group, only the %CM after loading were compared (one way ANOVA and Fisher’s LSD test). There was a statistically significant difference among groups (Figure 2). In terms of marginal performance, the groups could be grouped into three clusters: Cention Forte (57.9 ± 21.8) and Equia Forte HT (53.3 ± 30.71) were statistically equivalent and were the groups with the highest percentages of marginal adaptation; followed by Clearfil Majesty ES Standard (31.73 ± 10.95) and Tetric PowerFill (27.3 ± 15.2), which were equivalent; and finally Surefil one (8.1 ± 5.5) and Clearfil Majesty ES Flow Super Low (6.5 ± 5.6), which were equivalent. Note that the three clusters were different from one another (Figure 2).

### Internal Adaptation (Percentages of Continuous Internal Margins (%CM)) after Thermomechanical Loading (TML)

The internal adaptation analysis was run using a one-way ANOVA followed by Fisher’s post hoc test to check the existence of statistically significant differences among groups (Figure 3). The tests revealed that the means were statistically different, and in particular four clusters of groups could be detected (Figure 3): Cention Forte and Clearfil Majesty ES Standard; Tetric PowerFill and Equia Forte HT; Equia Forte HT and Clearfil Majesty ES Flow Super Low; and Surefil One. 

Representative SEM images from restoration margins of each group are shown in Figure 4.

## 4. Discussion

Treating children in the dental office can be challenging as they tolerate shorter treatment times, and their lack of cooperation might compromise the control of mouth humidity. In those cases, the restoration of decayed teeth with resin composites may show some disadvantages, as the stratification technique requires time, and the adhesion steps call for proper isolation. Therefore, this research study aimed to evaluate recently available restorative materials that could simplify the procedure to restore temporary decayed teeth, allowing shorter treatment times and a better handling of humid intraoral conditions. Based on the results, the null hypotheses tested, in that no difference would be detected in both marginal and internal adaptation of restorations of class II cavities with the different materials, had to be rejected.

Marginal adaptation, i.e., the percentages of continuous margins, immediately after restoration and after a fatigue test consisting of repeated cycles of thermal and mechanical loading, was the measured outcome as this parameter is considered one of the approaches to predict clinical behavior that is the closest to the clinical situation [10]). In addition, according to the FDI World Dental Federation clinical criteria, marginal adaptation is one among the six parameters that need to be assessed when judging on the functional properties of direct and indirect restorations [5]. In order to better simulate intra-oral conditions, a solution of phosphate-based saline (PBS) and horse serum was injected inside the pulp chamber of the teeth to simulate the presence of dentinal fluid [8,9]. All restored teeth went through a fatigue test consisting of 500 thermal cycles and 200,000 chewing cycles, which simulates approximately ten months under oral function. Gold-coated replicas made out of epoxy resin were procured from each tooth sample both before and after the fatigue tests to ensure a non-destructive evaluation. This allowed us to analyze the percentages of gap-free margins, i.e., marginal adaptation, both before and after simulated intra oral function, in order to evaluate if there was an effect of fatigue conditions on marginal quality. 

The analysis of internal adaptation, i.e., the percentages of gap-free adhesive interface inside the restoration, could only be analyzed after the fatigue test as the tooth samples had to be middle-cut to be able to expose the internal adhesive interface. 

The control group selected for this study was a highly filled glass ionomer, Equia Forte HT, as it is considered a gold standard for the restoration of temporary teeth due to its ease of handling, fluoride release, and chemical adhesion to Ca present within enamel and dentin, as well as its good tolerance to humidity [11,12].

The other groups consisted of an alkasite (dual-curing composite), bulk filling composites, and a self-adhesive composite, as these materials present additional advantages regarding ease of manipulation and saving of clinical time [3,13].

### 4.1. Cention Forte, an Alkasite-Based Dual Curing Composite

Initially, the manufacturer launched Cention N into the market, and then a recent version was launched, Cention Forte, which is almost the same composition except for the presence of a self-curing adhesive that is contained in the dedicated applicator that is activated when in contact with the primer, and the product is delivered in a capsule that is mixed electrically rather than manually. The highest results in marginal adaptation were obtained with this dual curing alkasite material. This material also showed the best percentage of continuous margins in terms of internal adaptation. However, it should be mentioned that some cohesive fractures within the material could be observed under SEM analysis. This finding was unexpected, as a previous study [14] reported that Cention Forte had Vickers values of more than 80 N/mm^2^, which is similar to the bulk-fill composites. This means that the material should be resistant enough against cohesive fractures. However, these authors did not put the specimens under fatigue. One possible cause for the cohesion fractures in our study could be the fact that Cention Forte was used in dual-curing mode, with only two times, 5 s each, of polymerization as proposed by the manufacturer. This was in order to follow our main purpose of simplifying the treatment. Furthermore, this dual polymerization also allowed a smaller contraction and better marginal adaptation, with chemical cure in the depth. It is possible that polymerization time was not enough, and that the material was not able to attain the desirable mechanical properties before water storage and fatigue test. This is a noteworthy aspect that should encourage further investigation as this study did not focus on the flexural strength of the materials.

### 4.2. Equia Forte HT, a Highly-Filled Glass Ionomer

In respect to Equia Forte HT, the percentages of continuous margins after thermomechanical loading were significantly higher than the other groups and equivalent to the alkasite material Cention Forte. It should be mentioned that Equia Forte HT could not be analyzed before loading because the coating layer, which is in fact proposed by the manufacturer, completely filled restoration margins, making the margin analysis before loading impossible. This coating might have infiltrated the margins and probably contributed to the improvement of marginal adaptation after thermomechanical loading. However, the analysis of internal adaptation revealed decreased %CM with respect to the groups restored with the alkasite material (Cention Forte) and the conventional composite (Clearfil Majesty ES Standard). This could be explained by the fact that the coating did not penetrate up to the internal adhesive interface so gaps were easily visible, but also because the adhesive interface between dentin and glass ionomer was not stress-resistant enough to withstand the fatigue test. Glass ionomers are known to poorly perform in stress-bearing areas, and their inferior mechanical properties could have directly affected the adhesive interface [15]. 

### 4.3. Clearfil Majesty ES Standard, a Conventional Composite

The group restored with conventional composite had lower values of marginal adaptation probably because no phosphoric acid (H_3_PO_4_) etching was used to condition enamel. This application protocol was used to reduce the treatment time by eliminating an additional application and rinsing step. Self-etching adhesives have been found to better interact with enamel when this substrate is selectively etched with H_3_PO_4_, even if it is during a short etching time of 10 s as elsewhere reported [16]. 

### 4.4. Clearfil Majesty ES Flow Super Low, a Flowable Composite

In respect to the flowable composite (Super Low Flow), a clear inferior performance was observed with respect to the conventional composite (Clearfil Majesty ES Standard) in terms of marginal integrity. This could be explained by the fact that flowable composite underwent a more important polymerization contraction, which is known to be related to a lower number of fillers [17]. The development of shrinkage forces is even further increased when the composite is applied in one single layer, as done in this study.

### 4.5. Surefil One, a Self-Adhesive Composite

The self-adhesive composite showed the lowest scores of marginal adaptation in both marginal and internal analyses. No adhesive system was used prior to its application, suggesting that the self-adhesive capacity of this material was insufficient to provide the restoration with a stress-resistant adhesive interface. These results could have been improved if an adhesive system was used together with this self-adhesive composite. Our results could not be compared to others reported in the literature for these materials, as studies have been focusing mainly on mechanical properties as flexural strength, like has been recently reported [15].

### 4.6. Tetric Powerfill, a Bulk-Fill Ultra-Fast Curing Composite

The bulk-fill ultra-fast curing composite showed intermediate values, both for marginal and internal adaptation. The percentages of continuous margins were lower compared to the study of Hamza et al. (2022) [18]. This could be due to the fact that only three seconds of polymerization was performed in our study compared to ten seconds in theirs, and probably the material was still insufficiently polymerized before the polishing phase. 

Finally, although this in vitro study attempted to simulate intraoral conditions as close as possible, some limitations should be mentioned: the shallow cavity preparations were often located on enamel. This means that adhesion could have been often established on enamel substrate, which is known to be less predictable unless phosphoric acid is used to condition this substrate [16]. Meanwhile, we avoided the use of H_3_PO_4_ for the etching of enamel so that treatment time could be reduced. In addition, scientific evidence is not clear about whether it is necessary to etch deciduous enamel or not [19]. Further studies are necessary in this respect.

## 5. Conclusions

This in vitro study was able to show, for the materials tested and under the above-mentioned restorative procedures, the highest results in terms of marginal and internal adaptation with alkasite dual-cured Cention Forte. From the clinical standpoint, this material could be considered an interesting restorative alternative for the restoration of deciduous teeth, especially when working under suboptimal clinical conditions in terms of humidity control and when easy handling and clinical time saving are necessary.

## Figures and Tables

**Table 1:** Detailed components of the materials used in the present study.

Material	Type	Manufacturer	Components	Polymerisation Mode
Equia Forte (batch number (BN): 190520A) + Equia Forte coating (self-adhesive, lightcuring resin coating) (BN: 181219A)	Glass Ionomer	GC, Tokyo, Japan	Powder: 95% strontium fluoro alumino-silicate glass, 5% polyacrylic acid Liquid: 40% aqueous polyacrylic acid Equia Forte Coat: 40–50% methyl methacrylate, 10–15% colloidal silica, 0.09% camphorquinone, 30–40% urethane methacrylate, 1–5% phosphoric ester monomer	Chemical + Lightcuring 20 s (coating)
Clearfil Universal Bond Quick (BN: 6B0214)	Adhesive	Kuraray Noritake Dental, Tokyo, Japan	10-Methacryloyloxydecyl dihydrogen phosphate (MDP), bisphenol A diglycidylmethacrylate (Bis-GMA), 2-hydroxyethyl methacrylate (HEMA), hydrophilic amide monomers, colloidal silica, silane coupling agent, sodium fluoride, dl-camphorquinone, ethanol, water	Lightcuring 10 s
Clearfil Majesty ES Super Low Flow (BN: 1N0030)	Superfilled composite resin (nanohybrid flowable composite)	Kuraray Noritake Dental, Tokyo, Japan	TEGDMA, hydrophobic aromaticdimethacrylate, silanated barium glass filler, pre-polymerized organic filler	Lightcuring 20 s
Clearfil Majesty ES standard (BN: CP0104)	Nanosuperfilled composite resin	Kuraray Noritake Dental, Tokyo, Japan	Silanated barium glass filler, prepolymerized organic filler, Bis-GMA, hydrophobic aromatic dimethacrylate, hydrophobic aliphatic dimethacrylate, dl-camphorquinon acceleratos, initiators, pigments (78 wt%, 66 vol%)	Lightcuring 20 s
Surefil One (BN: 2006000062)	Bulk fill self-adhesive composite	Dentsply Sirona, Konstanz, Germany	Aluminium-phosphor-strontium-sodium-fluoro-silicate glass, water, highly dispersed silicon dioxide, acrylic acid, polycarboxylic acid (MOPOS), ytterbium fluoride, bifunctional acrylate (BADEP), self-cure initiator, iron oxide pigments, barium sulfate pigment, manganese pigment, camphorquinone, stabilizer	Dual, lightcuring 20 s
Cention Forte (BN: Z01CR7) + Cention Forte primer	Bioactive powder-liquid filling material	Ivoclar Vivadent, Schaan, Liechtenstein	Powder: barium aluminum silicate glass, ytterbium trifluoride, isofiller, calcium barium aluminum fluorosilicate glass, calcium fluoro silicate glassLiquid: urethane dimethacrylate, tricyclodecan- dimethanol dimethacrylate, tetramethyl-xylylene diurethane dimethacrylate, polyethylene glycol 400 dimethacrylate, ivocerin, hydroxyperoxide	Capsule: Dual, 2 × 5 s Adhesive: selfcuring
Adhese Universal (BN: Y45234)	Universal adhesive	Ivoclar Vivadent, Schaan, Liechtenstein	Bis-GMA, HEMA, MDP, MCAP, decandiol dimethacrylate, dimethacrylate, ethanol, water, initiator, stabilizers, silicon dioxide	Lightcuring 3 s
Tetric Powerfill (BN: Z009FB)	Nanohybrid composite resin	Ivoclar Vivadent, Schaan, Liechtenstein	Bis-GMA, Bis-EMA, UDMA, PBPA, DCP, β-allyl sulfone (filler mass 76–77%, vol 53–54%)	Lightcuring 3 s

## Data Availability

The original contributions presented in the study are included in the article, further inquiries can be directed to the corresponding authors.
